# Muscle wasting in cancer cachexia: Mechanisms and the role of exercise

**DOI:** 10.1113/EP092544

**Published:** 2025-03-30

**Authors:** Zoe P. Libramento, Louisa Tichy, Traci L. Parry

**Affiliations:** ^1^ Department of Kinesiology University of North Carolina Greensboro Greensboro North Carolina USA

**Keywords:** atrophy, cancer cachexia, exercise

## Abstract

Cancer cachexia (CC) is a multifactorial disease marked by a severe and progressive loss of lean muscle mass and characterized further by inflammation and a negative energy/protein balance, ultimately leading to muscle atrophy and loss of muscle tissue. As a result, patients experiencing cachexia have reduced muscle function and thus less independence and a lower quality of life. CC progresses through stages of increasing severity: pre‐cachexia, cachexia and refractory cachexia. Two proposed underlying mechanisms that drive cancer‐induced muscle wasting are the autophagy–lysosome and ubiquitin–proteasome systems. An increase in autophagic flux and proteolytic activity leads to atrophy of both cardiac and skeletal muscle, ultimately mediated by tumour or immune‐secreted inflammatory cytokines. These pathways occur at a basal level to maintain cellular homeostasis; therefore, it is the overactivation of the pathways that leads to muscle atrophy. Recent evidence demonstrates the ability of aerobic and resistance training to restore these pathways to their basal levels. The mechanism is not yet understood, and more research is needed to determine exactly how exercise influences each pathway. However, exercise has great promise as a therapeutic strategy for CC because of the evidence for it preserving muscle mass and function, and attenuating protein degradative pathways. The extent to which exercise affects the ubiquitin–proteasome and autophagy–lysosome systems is determined by the frequency, intensity and duration of the exercise protocol. As such, an ideal exercise prescription is lacking for individuals with CC.

## Abstract (highlights)

- **What is the topic of this review?**
  This review explores emerging molecular protein degradation and inflammation mechanisms of cancer cachexia (CC) and highlights how exercise regulates these pathways and thus can be leveraged as an effective adjuvant therapy for CC.
- **What advances does it highlight?**
  Exercise regulates the autophagy–lysosome and ubiquitin–proteasome systems to preserve muscle mass and improve muscle function. Exercise has been shown to be a valuable strategy for mitigating CC‐mediated cardiac and skeletal muscle wasting.

## 1 INTRODUCTION

From the Greek words *kakos*, meaning ‘bad’, and *hexis*, meaning ‘condition’, cachexia is a multi‐factorial condition that causes progressive muscle loss with or without adipose tissue loss (Fearon et al., 2011). This progressive wasting syndrome is characterized by a loss of lean muscle mass, systemic inflammation, and metabolic alterations (Fearon et al., 2011). While cachexia can occur in many chronic diseases, such as AIDS, chronic obstructive pulmonary disease or heart disease, it is most often associated with cancers, specifically during advanced stages of cancer (Baker Rogers et al., 2024). Individuals with cancer cachexia (CC) have a poorer prognosis, reduced responses to anti‐cancer therapies, reduced physical function and an overall lower quality of life (Rausch et al., 2021). The hallmark feature of cachexia is skeletal muscle wasting, which ultimately results in severe weight loss. Two proposed underlying mechanisms that are upregulated in atrophying muscles, the autophagy–lysosome and ubiquitin–proteasome systems, drive cancer‐induced muscle wasting (Rausch et al., 2021). Excessive protein degradation in the muscle causes a negative protein balance and consequent loss of muscle tissue. Currently, there are no standard treatment options for cachexia. Physical activity is a promising non‐pharmacological intervention that has a strong potential to prevent and protect against cancer‐induced cachexia by regulating inflammation as well as intracellular protein degradation systems. As a cost‐effective and accessible treatment strategy, exercise is a valuable tool preserving functional capacity and quality of life during CC.

## 2 CANCER CACHEXIA

### 2.1 Definition, symptoms and statistics

CC is a multifactorial syndrome marked by severe and progressive loss of lean muscle mass that may or may not be accompanied by adipose tissue loss. CC is characterized by systemic inflammation, negative energy and protein balance, and a loss of lean muscle mass which cannot be reversed by nutritional support alone (Fearon, 2011). Approximately 50% of all cancer patients will experience cachexia. However, the incidence of CC differs across cancer types: patients with gastric and pancreatic cancer experience the highest frequency of muscle wasting, followed by lung, prostate and colon cancer (Tisdale, 2005). Typically, cancer types associated with changes in metabolism or mutations in metabolic genes are most heavily associated with CC development and rapid progression (Karuppannan et al., 2024; Tayek, 1992; Tisdale, 2009). Widespread muscle wasting results in a loss of functional capacity and leads to a drastic decrease in quality of life. While diagnostic tools and criteria are still not fully developed, the ability to identify high‐risk of patients with metabolic cancers could improve clinical outcomes. This highlights the importance of CC assessment and individually tailored treatment approaches based on cancer type and severity of CC symptoms.

The consensus definition of CC is a metabolic syndrome with a loss of 5% or more of body weight in 6 months (Evans et al., 2008; Fearon et al., 2011). The hallmark features of this syndrome are the progressive loss of lean muscle mass leading to a significant loss of body weight, muscle function impairment and fatigue. Furthermore, Evans et al. (2008) describe abnormal biochemistry as a key component of CC, including inflammation, insulin resistance and protein imbalance, resulting in the loss of muscle proteins. While skeletal muscle loss is the most obvious and devastating consequence of CC, cardiac tissue and other organs can experience atrophy as well, with heart and respiratory failure contributing to about 20% of cancer‐related deaths (Ni & Zhang, 2020). Individuals suffering from CC experience accelerated weight loss, reduced muscle strength and function, increased fatigue, and anorexia, all of which can lower an individual's independence and quality of life – and ultimately survival.

### 2.2 Staging

Cancer‐associated cachexia exists on a continuum divided into three stages: pre‐cachexia, cachexia and refractory cachexia. Pre‐cachexia is the earliest stage and is defined as less than 5% loss of body weight, which may be preceded by metabolic changes such as glucose intolerance and anorexia (Fearon et al., 2011). Treatment for individuals diagnosed with pre‐cachexia generally focuses on preventative measures, such as exercise and nutritional supplementation, and the individual's condition is continuously monitored (Fearon et al., 2011; Ni & Zhang, 2020). As an individual's condition progresses, a more significant loss of lean muscle marks the transition into the cachexia stage. It is important to note the mechanism of cachexia differs from that of sarcopenia (age‐related muscle loss). Sarcopenia is generally associated with reductions in muscle protein synthesis, while cachexia is characterized more predominantly by excessive muscle protein breakdown (Argilés et al., 2003). Furthermore, sarcopenia is not usually associated with weight loss, as body fat is gained throughout the development of sarcopenia and thus the term ‘sarcopenic obesity’ is used (Wiedmer et al., 2021). However, some preclinical studies observed decreases in protein synthesis as well as increases in protein breakdown during CC (Brown et al., 2018; Smith & Tisdale, 1993)

Cachexia entails a loss of 5% or more of body weight over 6 months. If an individual's baseline body mass index is less than 20 kg/m^2^ then a loss of 2% or greater is a sufficient criterion for the diagnosis of cachexia (Fearon et al., 2011). An individual with cachexia exhibits progressive loss of lean muscle mass and strength, and thus decreased functional capacity (Fearon et al., 2011). This stage requires multi‐modal treatment, including physical therapy and nutritional counselling.

The last stage is refractory cachexia, the manifestation of extreme muscle wasting. In refractory cachexia, a loss of 15–20% or more of body weight is typically seen in 6 months or less (Baker Rogers et al., 2024). These individuals are no longer responsive to anticancer therapies and weight loss management is no longer feasible during this stage. Palliative care becomes the focus as life expectancy at this stage is <3 months (Fearon et al., 2011). Despite having established criteria, CC remains poorly diagnosed and patients often progress to later stages of the disease before intervention takes place. Early diagnosis strategies, such as biomarkers, and effective therapies have yet to be discovered.

## 3 PROTEIN DEGRADATION SYSTEMS

Under normal conditions, muscle mass is carefully sustained by maintaining rates of muscle protein synthesis and protein degradation within the cell. Both processes occur simultaneously, and therefore if the rate of protein degradation exceeds the rate of synthesis, over time muscle atrophy will occur. The imbalance of protein degradation and synthesis is a key characteristic of CC, and two protein‐degradation systems have been implicated in cancer‐induced muscle wasting: the ubiquitin–proteasome and autophagy–lysosome systems (Jamart et al., 2012).

### 3.1 The ubiquitin–proteasome system

The ubiquitin–proteasome system (UPS) is a protein degradation system in which a target protein is tagged with several ubiquitin molecules, recognized by a 26S proteasome, and subsequently degraded into smaller peptides (Sandri, 2013).The UPS machinery works by covalently attaching a chain of ubiquitin molecules to the target protein and is coordinated by an enzymatic cascade of atrogenes consisting of E1 (ubiquitin‐activating), E2 (ubiquitin‐conjugating) and E3 (ubiquitin ligase) enzymes. Ubiquitin must first be activated by the E1 enzyme, after which it is conjugated with the E2 enzyme. With the help of an E3 ligase, E2 attaches ubiquitin to the protein of interest. Multiple ubiquitin molecules will be attached to this protein until the proteasome recognizes it. The 26S proteasome involved is a large, multi‐catalytic protease that identifies this ubiquitinated protein and degrades it into smaller peptides. In doing so, the UPS rids the cell of misfolded, damaged, or excess proteins, as their accumulation may impede normal cell function. Thus, the UPS is responsible for maintaining cellular protein homeostasis and energy homeostasis, and the resulting peptides can be used in other metabolic processes (i.e., energy production) (Cohen‐Kaplan et al., 2016).

Preclinical studies have shown the UPS is significantly upregulated during CC and contributes to the associated muscle wasting (Pigna et al., 2016; Tanaka et al., 2019). There is direct evidence that an increase in UPS markers correlates with an increase in muscle protein breakdown (Khal et al., 2005; Pang et al., 2023; Yuan et al., 2015), though there is limited evidence that suggests otherwise (Hughes et al., 2023). Muscle RING‐finger protein‐1 (MuRF1) and muscle atrophy F‐box (MAFbx; aka Atrogin‐1) are two skeletal muscle‐specific ubiquitin ligases, responsible for tagging the selected substrate with ubiquitin. Previous studies have identified them as key markers of skeletal and cardiac muscle atrophy (Jamart et al., 2012; VanderVeen et al., 2017). Specifically, MuRF1 ubiquitinates muscle contractile and structural proteins such as troponin, myosin heavy chains and actin (Sandri, 2013). The loss of these structural proteins results in myofibre and muscle atrophy.

### 3.2 The autophagy–lysosome system

The term ‘autophagy’ is derived from the Greek words *auto* and *phagein*, its literal translation being ‘eating oneself’ (Halling & Pilegaard, 2017). The autophagy–lysosome system is a bulk protein degradation that discards damaged, old or unneeded cellular material by engulfing it and recycling its components back into the cytoplasm to maintain cellular homeostasis (Gunadi et al., 2021). Autophagic flux refers to the entire process of autophagy, from the formation of the phagophore to the degradation of the material. During times of cellular stress, autophagic flux is upregulated to sustain proper cellular function. On the contrary, the downregulation of autophagy occurs during times of nutrient abundance and is modulated via mammalian target of rapamycin (mTOR) (Halling & Pilegaard, 2017). There are three types of autophagy: microautophagy, macroautophagy and chaperone‐mediated autophagy (Parzych & Klionsky, 2014). Of these three distinct types, macroautophagy is the best characterized, includes selective autophagy (i.e., mitophagy – the breakdown of mitochondria by autophagic pathways), and will be discussed in this review, hereafter referred to as ‘autophagy’ (Tanida, 2011). This degradative system is tightly regulated and responds to stressors such as fasting, ageing, disease and cancer. In this way, the autophagy–lysosome system is prone to a variety of perturbations – particularly excessive upregulation during CC.

#### 3.2.1 Autophagic flux

During autophagy, a double‐membrane vesicle, known as a phagophore (or isolation membrane), is formed *de novo* around a ubiquitinated protein of interest. The membrane elongates and ultimately generates a spherical autophagosome, which wraps completely around the cytosolic material until it fuses with itself, thus fully enclosing the material, at which point it is referred to as a mature *autophagosome* (Parzych & Klionsky, 2014). The autophagosome fuses with a lysosome to form an *autolysosome*. The cargo of the autolysosome is exposed to the acidic lumen of the lysosome and inevitably becomes degraded. The remaining components (consisting of amino acids, carbohydrates and lipids) can be recycled back into the cell to be used to build new material or to produce energy.

The process of autophagy is under meticulous control because of its important role in maintaining protein turnover in the cell. The induction of autophagy and formation of the autophagosome is driven by a cohort of proteins known as autophagy‐related (ATG) proteins (Halling & Pilegaard, 2017). Both mTOR and AMP‐activated protein kinase (AMPK) play a significant role in the regulation of autophagic flux by influencing these ATG proteins (Pigna et al., 2016). mTOR is a major controller of cell growth and survival via phosphoinositide 3‐kinase/AKT/mTOR pathway. However, it also plays a major role in regulating autophagy by influencing the ULK complex, which is involved in phagophore formation (Pigna et al., 2016). Under nutrient‐rich conditions, mTORC1 is activated and phosphorylates the ULK complex, thus deactivating this complex and downregulating autophagy. However, when nutrients are limited, mTORC1 is inactive, and therefore the ULK complex is dephosphorylated and activated, and autophagy is upregulated.

Similarly, AMPK can regulate autophagic flux. AMPK is an energy‐sensing molecule that plays an important role in energy homeostasis by activating catabolic processes and inhibiting anabolic processes during times of cellular need. During low‐energy conditions, AMPK suppresses energy‐demanding cellular processes and induces autophagy by inhibiting mTOR activity and increasing expression of FOXO1, a major regulator of autophagy and the UPS (Pigna et al., 2016; Tanaka et al., 2019). AMPK and mTOR regulate autophagic flux depending on the body's energetic demands. The presence of cancer increases energy demands as tumour cells compete for nutrients, and therefore similar to the UPS, the autophagy–lysosome system is implicated in cancer‐induced muscle wasting during cachexia (Cosper & Leinwand, 2011; Pigna et al., 2016; Rausch et al., 2021).

## 4 CC: MUSCLE DYSFUNCTION AND WASTING

It is widely accepted that increased muscle protein degradation is a major contributor to muscle loss associated with CC. However, changes in muscle protein synthesis are also observed during CC (Brown et al., 2018; Smith & Tisdale, 1993). The identification of the autophagy–lysosome system and UPS as possible major mechanisms underlying muscle atrophy in CC is significant as it provides an opportunity for more targeted therapeutic options for cachexia treatment. CC can potentially cause atrophy of both skeletal and cardiac muscle tissue, although more research is needed to clarify the differences between these two tissues.

### 4.1 Skeletal muscle atrophy

The maintenance of muscle mass relies on a balance of protein degradation and synthesis. A disruption of this balance can result in the loss of skeletal muscle mass, which is the most prominent physical manifestation of cancer‐induced cachexia (Evans et al., 2008). Consequently, cachectic individuals struggle with reduced muscle strength, fatigue, anorexia and biochemical abnormalities such as inflammation and insulin resistance (Evans et al., 2008). In humans, skeletal muscle comprises approximately 30–40% of total body weight and is essential for body movement, thermoregulation and energy metabolism (Daou, 2020). Additionally, skeletal muscle acts as the body's largest store of amino acids, essential for various cellular functions, and contributes to glucose uptake and insulin sensitivity, playing a role in maintaining metabolic homeostasis (VanderVeen et al., 2017). As a result, skeletal muscle atrophy is of significant concern as it results in serious functional decline, frailty and a lower quality of life.

**FIGURE 1:**
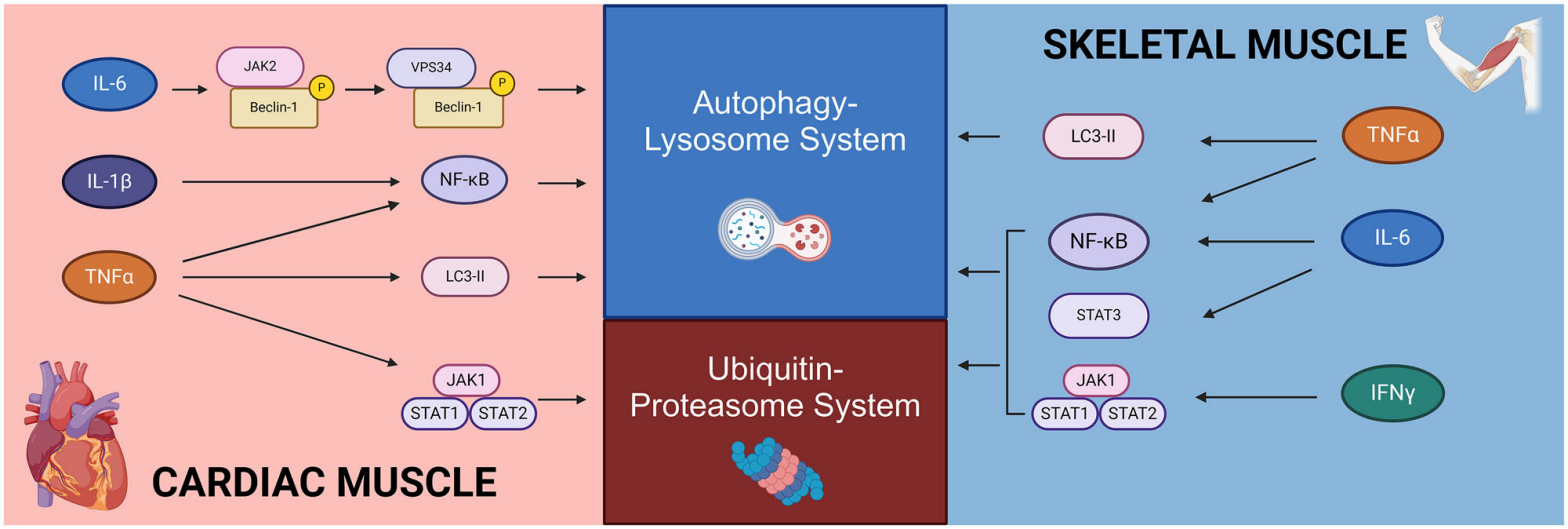
Inflammatory pathways promote muscle protein degradation pathways. In the heart and skeletal muscle inflammatory cytokines IL‐1β, IL‐6, TNFα and IFNγ interact with the autophagy–lysosome system and the UPS to maladaptively upregulate protein degradation. This in turn leads to cardiac and skeletal muscle wasting, overall body fatigue, and poor quality of life. IFNγ, interferon‐γ; JAK, Janus kinase; IL, interleukin; NF‐κB, nuclear factor‐κB; STAT, signal transducer and activator of transcription; TNFα, tumour necrosis factor‐α; UPS, ubiquitin–proteasome system. Created with BioRender.com.

It is well‐established that CC is an inflammatory syndrome, with cytokines such as tumour necrosis factor‐α (TNFα), interleukin (IL)‐6, and interferon‐γ (IFNγ) playing important roles in its development (Rausch et al., 2021). TNFα, originally known as *cachectin*, is a major inflammatory mediator responsible for regulating the body's pro‐inflammatory response, cell proliferation, cell survival and cell differentiation (Daou, 2020). This cytokine is also implicated in CC and is proposed to induce proteolytic pathways that lead to muscle atrophy. Studies have reported a TNFα‐induced increase in autophagy markers, such as LC3 (light chain 3), p62 and Beclin‐1, in the skeletal muscle of tumour‐bearing animals (Chu et al., 2017; Hentilä et al., 2019; Penna et al., 2013). Additionally, TNFα promotes additional catabolism by upregulating proteolysis (i.e., UPS) via nuclear factor‐κB (NF‐κB) (Figure 1) (Rausch et al., 2021; VanderVeen et al., 2017).

Another pro‐inflammatory cytokine, IL‐6, is also implicated in skeletal muscle wasting during CC (Carson & Baltgalvis, 2010; Daou, 2020). Recently, IL‐6 has emerged as a primary mediator of tumour growth and metastasis and is considered a central player in CC (Daou, 2020). In a cachectic mouse model, overexpression of IL‐6 in skeletal muscle is associated with severe weight loss and greater tumour burden (Baltgalvis et al., 2008). Skeletal muscle acts as a target of IL‐6, where it suppresses mTORC1 activity (upregulating autophagy), and downstream signal transducer and activator of transcription (STAT)‐3 and NF‐κB induce both the UPS and the autophagy–lysosome system (Figure 1) (Narsale & Carson, 2014; White et al., 2013). Similarly, IFNγ activates the Janus kinase (JAK)/STAT pathways ultimately leading to protein degradation and muscle atrophy (Figure 1) (Schroder et al., 2003). Therefore, it is clear that, during CC, an increase in autophagic flux and proteolytic activity leads to atrophy of both cardiac and skeletal muscle, ultimately mediated by tumour or immune‐secreted inflammatory cytokines (Gunadi et al., 2021; Penna et al., 2013).

Most of these studies do not differentiate the subjects based on the stage of cachexia Therefore, there is little evidence on how these markers change specifically across the CC continuum (from early‐stage pre‐cachexia to late‐stage refractory cachexia). While CC is diagnosed by assessing muscle mass alongside inflammatory markers and metabolic changes, these measures are not usually collected in clinical settings, nor are there agreed‐upon standards for diagnosis. Due to the lack of clear functional cutoffs, discrete biomarkers, and proper surveillance, data are often insufficient for early diagnosis and intervention.

### 4.2 Cardiac muscle atrophy

The wasting of cardiac tissue during CC, known as cancer‐mediated cardiac cachexia (hereafter referred to as ‘cardiac cachexia’), leads to cardiac complications, which are the leading cause of death in cancer patients (Rausch et al., 2021). Research shows cardiac wall thickness significantly decreases during cardiac cachexia, indicated by a decrease in left ventricular posterior wall thickness, which contributes to a reduction in fractional shortening and ejection fraction, measured with echocardiography (Belloum et al., 2017). Thus, maintaining adequate cardiac wall thickness is important as it correlates directly with efficient cardiac function, and atrophy of the heart (e.g., thinning of cardiac walls) leads to a host of cardiac abnormalities that manifest as fatigue, shortness of breath, loss of appetite and reduced exercise tolerance (Murphy, 2016).

Similar to the induction of cachexia in skeletal muscle, the pathogenesis of cardiac cachexia is associated with pro‐inflammatory cytokines such as IFNγ, TNFα, IL‐1 and IL‐6 (Daou, 2020). Pro‐inflammatory cytokines TNFα, IL‐1 and IL‐6 activate the JAK/STAT pathway, which results in an increase in protein breakdown and a decrease in protein synthesis (Daou, 2020). High levels of IL‐6 indicate inflammation and can lead to systolic dysfunction, ventricular dilatation and cardiomyocyte apoptosis, thus promoting the development of heart failure (Su et al., 2021). More specifically, IL‐6 induces autophagy by promoting the interaction between JAK2 and Beclin‐1, thus leading to the phosphorylation of Beclin‐1 and subsequent induction of autophagy (Hu et al., 2021). During CC, autophagic markers such as LC3‐II, Beclin‐1 and cathepsin L have been identified and their expression is upregulated in the heart, indicating increased lysosomal degradation (Cosper & Leinwand, 2011). In a preclinical model, Parry et al. (2022) found an increase in cardiac expression of Beclin‐1 and p‐FOXO1, which is known to regulate both autophagy and the UPS. Furthermore, Parry & Willis (2016) found upregulation in cardiac ubiquitin ligases during cardiac cachexia. The overactivation of the autophagy–lysosome system increases protein degradation, shifting the balance away from muscle protein synthesis and toward excessive muscle protein degradation to ultimately drive cardiac atrophy and remodelling. Although specific mechanisms vary between skeletal and cardiac muscle (Figure 1), increased inflammation and dysfunctional metabolism play a large role in inducing this muscle wasting during CC.

## 5 EXERCISE AS A THERAPEUTIC INTERVENTION FOR CC

Engaging in regular exercise not only aids in cancer prevention but also significantly lowers the chances of relapse and enhances survival rates among cancer patients (McTiernan et al., 2019). Physical activity has been recently identified as a protective mechanism against cancer incidence, and as we broaden our knowledge of exercise's benefits for cancer, its utility in clinical settings (e.g., cancer rehab) is becoming increasingly recognized for its value. For cancer patients suffering cachexia, recent data show that exercise may be a critical treatment strategy for slowing (or preventing) CC progression. Cachexia recommendations highlight the importance of a proactive approach in the early stages of development with a focus on maintaining physical fitness to reduce the rate of muscle wasting (Maddocks et al., 2011). Exercise holds great promise as a therapeutic strategy for CC because of its ability to modulate protein degradation and protein synthesis and for its anti‐inflammatory effects.

### 5.1 Exercise and the UPS in CC

Exercise temporally affects proteasomal activity to meet the increased energy demand during exercise to sustain activity (Jamart et al., 2012). This can be seen in healthy individuals, where acute bouts of both resistance training and aerobic exercise transiently increase skeletal muscle Atrogin‐1 and MuRF1 levels during recovery (Louis et al., 2007). Furthermore, eccentric contractions are associated with increased ubiquitination of muscle proteins (Stupka et al., 2001; Thompson & Scordilis, 1994). During CC, however, the normal function of the UPS is profoundly impacted. Therefore, it is unclear how the UPS might be impacted when under stress via resistance training and/or aerobic training in addition to CC.

Recent preclinical studies have explored how the UPS is affected by aerobic exercise training in CC (Table 1) (Pigna et al., 2016; Tanaka et al., 2019). Pigna et al. investigated the effects of 19 days of voluntary wheel‐running in C26‐inoculated mice, while Tanaka et al. used a low‐intensity treadmill protocol on AH130 Wistar rats for 10 days. In both studies, consistent low‐intensity exercise resulted in a notable downregulation of UPS activity, shown by a significant decrease in skeletal muscle Atrogin‐1 and MuRF1 protein expression (Pigna et al., 2016; Tanaka et al., 2019). Furthermore, because both exercise protocols were of low intensity, exercise intensity may also have a large impact on UPS modulation. Little research has been done using high‐intensity exercise protocol and its influence on the UPS. More research is needed on how aerobic exercise training protocols of different intensities may affect the cellular biochemistry, specifically autophagic and proteolytic degradation.

**TABLE 1:** Exercise effects on protein degradation systems in preclinical CC models.

Preclinical model	Muscle type	Exercise type	Exercise intensity	Exercise duration	Mode of exercise	Protein degradation system	Reference
Cardiac Muscle							
Fisher 344 rats, MatBIII mammary gland tumour	Heart	AT	N/A	6 weeks	Voluntary wheel running	Autophagy ↓ LC3‐II	Parry & Hayward (2018)
Balb/c mice, C26 colon tumour	Heart	AT	60% maximum speed	60 min, 5×/week for 45 days	Treadmill running	Autophagy ↓ BNIP3	Fernandes et al. (2020)
Copenhagen rats, Dunning R‐3327 prostate adenocarcioma	Heart	AT	>75% maximal aerobic capacity	60 min, 5×/week for 20 days	Treadmill running	Atrogin1 expression same between groups	Baumfalk et al. (2021)
C57/BL6 mice, LLC lung tumour	Heart	AT	65% ${\dot{V}}_{O_{2}\max}$	60 min/day, 5×/week, for 4 weeks	Treadmill running	Autophagy ↓ Beclin‐1 ↓ P‐FOXO1 ↓ MyD88	Parry et al. (2022)
Skeletal Muscle							
Balb/c mice, C26 colon tumour	Tibialis anterior	AT	N/A	5–19 days	Voluntary wheel running	UPS ↓ Atrogin1 ↓ MuRF1 Autophagy ↓ LC3‐II/LC3‐I ratio	Pigna et al. (2016)
Balb/c mice, C26 colon tumour	Tibialis anterior	AT + RT	RT: light‐moderate AT: light	4×/week for 4 weeks	Motorized wheel running Resistance ladder climbing	Autophagy ↓ LC3‐II/LC3‐I ratio	Ranjbar et al. (2019)
Balb/c × C57/BL6 mice (overexpressing muscle specific PGC‐1α), C26 colon tumour	Gastrocnemius, tibialis anterior	AT	Light‐ moderate	45 min, 3×/week for 12 days	Treadmill running	Autophagy ↓ LC3‐II/LC3B‐I ratio ↓ LC3‐II ↓ p62/SQSTM1 but NS For healthy mice: ↑ LC3‐I	Ballarò et al. (2019)
Swiss mice, Ehrlich breast carcinoma	Soleus and extensor digitorum longus	RT	NS	3×/week for 4 weeks	Resistance ladder climbing	Autophagy ↓ LC3B‐II ↓ Belcin1 ↓ p62	Testa et al. (2022)
Wistar rats, AH130 liver tumour	Plantaris	AT	Light	30 min/session, 8 sessions across 10 days	Treadmill running	UPS ↓ Atrogin1 ↓ MuRF1	Tanaka et al. (2019)

Abbreviations: AT, aerobic training; CC, cancer cachexia; N/A, not applicable; NS, not specified; RT, resistance training.

Furthermore, resistance training has also been observed to attenuate cachectic symptoms and muscle atrophy. Testa et al. (2022) demonstrated the effectiveness of a 28‐day resistance training protocol (ladder‐climbing for mice) in attenuating degradative pathways, where it was seen that resistance training prevented an increase in FOXO1 and FOXO3 gene expression as well as Atrogin‐1 protein expression. This study also demonstrated that resistance training prevented the elevation of IL‐6 levels in skeletal muscle, thus attenuating STAT3 phosphorylation and preventing muscle wasting. As stated before, IL‐6 signals through STAT3 to ultimately trigger UPS activity. These data suggest that chronic resistance exercise training can cause beneficial adaptations that decrease protein degradation, and these adaptations affect pathways at the transcription level. A primary goal of CC treatment is to specifically target the pathways underlying muscle wasting. If chronic exercise training can decrease ubiquitin–proteasome activity and reduce muscle atrophy, then exercise can be harnessed as a therapy for cachectic cancer patients.

### 5.2 Exercise and the autophagy–lysosome system in CC

Autophagy is required at a basal level in skeletal muscle to maintain muscle mass. During exercise, autophagy is important in clearing damaged proteins and organelles to maintain muscle function and generate energy substrates to sustain activity (Escobar et al., 2018). Temperature and pH changes, and oxidative and mechanical stress all contribute to increased protein degradation during acute bouts of exercise. Therefore, autophagy's role during exercise is of high importance to maintain efficient cellular activity and health. As seen with the UPS, acute bouts of exercise have been shown to transiently increase the autophagy–lysosome system in preclinical studies, with an increase in skeletal muscle expression of LC3‐I and LC3‐II in healthy mice after a single bout of treadmill running (He et al., 2012; Pagano et al., 2014). In humans, ultra endurance runners that ran for 24 h on a treadmill showed increased expression levels of LC3‐II, *Atg12* and *Atg5 (autophagy‐related genes)* (Table 2) (Jamart et al., 2012).

**TABLE 2:** Exercise effects on protein degradation systems in clinical studies.

Clinical model	Muscle type	Exercise type	Exercise intensity	Exercise duration	Mode of exercise	Protein degradation system	Reference
Healthy, non‐smoking males and females	Vastus lateralis	RT	120% of 1‐RM	Leg press: 3 sets × 12 reps Leg extension: 10 sets × 10 reps	Weight machines: Eccentric, single‐leg muscle action using leg press + knee extension	UPS ↑ Ubiquitin‐conjugated enzymes	Stupka et al. (2001)
Untrained, apparently healthy males and females	Biceps	RT	NS	5 sets × 53 reps	Biceps curl	UPS ↑ Ubiquitin levels	Thompson & Scordilis (1994)
Healthy, well‐trained males	Vastus lateralis	AT	NS	24 h	Running	Autophagy ↑ LC3b ↓ p‐Akt ↓ p‐mTOR UPS ↑ MuRF1	Jamart et al. (2012)
Healthy, physically active males and females	Vastus lateralis, gastrocnemius	AT + RT	RT: 70% 1‐RM AT:75% ${\dot{V}}_{O_{2}\max}$	RTL 3 sets × 10 reps AT: 30 min	RT: leg extension machine AT: running	UPS ↑ Atrogin‐1, MuRF1, and FOXO3a	Louis et al. (2007)

Abbreviations: 1‐RM; one repetition maximum; AT, aerobic training; N/A, not applicable; NS, not specified; RT, resistance training.

Despite the upregulation of autophagic activity after an acute bout of exercise, chronic physical activity is shown to have a different effect on basal autophagy levels (Ballarò et al., 2019; Grumati et al., 2011; Parry & Hayward, 2018; Pigna et al., 2016; Ranjbar et al., 2019; Testa et al., 2022). When exercise is continued for several days or weeks, overall autophagic activity is reduced, suggesting that autophagic flux is regulated by long‐term exercise adaptation. This may additionally be impacted by the presence of disease, such as cancer and CC. For example, in Ballarò et al.’s study, C26 tumour‐bearing mice performed treadmill running 3 days a week for 12 days after tumour cell inoculation. At the end of the study, the mice were seen to have reduced levels of LC3‐II and a reduced LC3‐II/LC3‐I ratio in their skeletal muscle compared to sedentary mice, suggesting that autophagy is downregulated with exercise during tumour‐bearing (Ballarò et al., 2019). Similarly, Pigna observed a decrease in both LC3‐II and p62 in C26 mice after 19 days of voluntary wheel running (Pigna et al., 2016). By downregulating autophagic activity during CC, protein degradation is reduced, and muscle atrophy is decreased. Furthermore, exercise prevents the downregulation of muscle protein synthesis via deactivation of mTOR, thus contributing to the preservation of muscle mass (Tanaka et al., 2019). This reduction in protein degradation, coupled with the increase in synthesis, promotes muscle growth, or at least maintains skeletal muscle mass in cachectic mice.

There are fewer data available for the benefits of resistance training for cancer‐induced muscle wasting. Testa et al. (2022) determined that resistance training reduced autophagic markers such as Beclin‐1, p62 and LC3 in breast cancer mice. Ranjbar et al. (2019) used a combined aerobic and resistance training protocol for his C26‐inoculated mice, which resulted in a lower skeletal muscle LC3‐II/LC3‐I ratio. Therefore, resistance training may be a treatment option for CC; however more preclinical research is needed to understand its influence on muscle atrophy.

On the same note, there are also very few data available on the impact of exercise on autophagy specifically in cachectic hearts. In a study conducted by Parry & Hayward (2018), LC3‐II levels were found to be reduced in the heart of tumour‐bearing rats after 6 weeks of voluntary wheel running, suggesting that cardiac autophagic activity changes with adaptation to long‐term exercise, as seen with skeletal muscle. Parry et al. (2022) also showed that preconditioning exercise can reduce overall autophagic activity, as seen with lower Beclin‐1 levels in tumour‐bearing mice after an 8‐week treadmill protocol. These data show that the body's autophagic response to exercise can be determined by the length of exercise intervention as well as the timing of exercise intervention within the cancer continuum, thus providing a distinction between autophagic adaptation to exercise and acute autophagic response to exercise.

More research is needed to understand the exact mechanisms of how chronic aerobic and resistance exercise influences these systems in cachexia models. There is an evident benefit to engaging in regular physical activity for the cancer patient; however, understanding the underlying mechanisms will allow for the creation of more specific targeted and synergistic therapies for CC.

### 5.3 Exercise and inflammation

**FIGURE 2:**
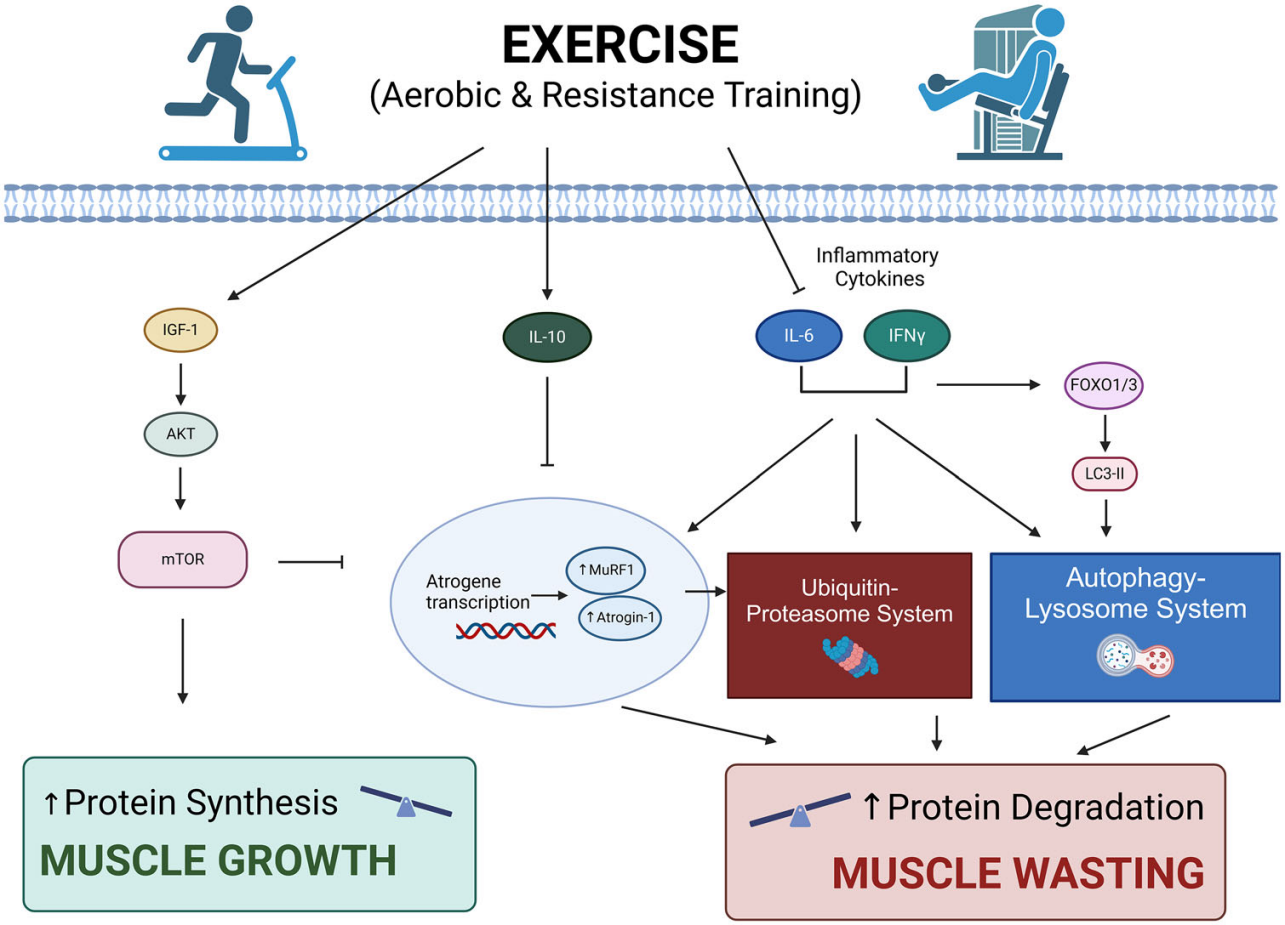
Exercise counteracts CC's muscle wasting effects. Exercise stimulates muscle growth via the IGF‐1/AKT/mTOR pathway and this inhibits protein degradation pathways. Exercise also has anti‐inflammatory effects that upregulate the anti‐inflammatory IL‐10 as well as downregulate pro‐inflammatory cytokines IL‐6 and IFN‐γ. Together exercise's anti‐inflammatory effect downregulates atrogene (MuRF1, Atrogin‐1) transcription, the ubiquitin‐proteasome system, and the autophagy‐lysosome system. The net effect tips the balance toward protein synthesis and away from protein degradation to ultimately protect against CC mediated cardiac and skeletal muscle wasting. CC, cancer cachexia; IFNγ, interferon γ; IL, interleukin; mTOR, mammalian target of rapamycin; MuRF1, muscle RING‐finger protein‐1; UPS, ubiquitin–proteasome system. Created with BioRender.com.

Inflammation is a significant factor in the onset and development of cancer, and CC is currently categorized as an inflammatory condition. Furthermore, it is clear that inflammatory cytokines are involved in the pathogenesis of skeletal and cardiac muscle wasting during CC (Gielen et al., 2003). Therefore, the mitigation of inflammation may play a major role in slowing cancer and CC progression (Fearon et al., 2011). Alongside its ability to preserve muscle mass and functional capacity, aerobic exercise training is known to have anti‐inflammatory effects, indicated by an increase in anti‐inflammatory cytokines, such as IL‐10 (Figure 2) (Belloum et al., 2017; Gould et al., 2013). Therefore, the integration of exercise training into cancer prevention and treatment strategies is critical for optimizing patient outcomes. Exercise can increase muscle growth and strength, increase cellular oxidative capacity, decrease oxidative stress, decrease inflammation, and protect against muscle atrophy and damage.

Circulating levels of inflammatory cytokines increase during and after exercise due to the physiological stress of activity. However, chronic aerobic exercise reduces overall inflammation despite the acute increase in inflammatory cytokines during and after exercise (Alves et al., 2015). Exercise used as either a preventative measure or a treatment can prevent or delay the progression of cancer‐induced muscle wasting, largely due to decreasing systemic inflammation. Furthermore, aerobic exercise is associated with higher levels of anti‐inflammatory cytokine IL‐10, which can further reduce systemic inflammation, increase protein synthesis and decrease protein degradation (Tichy & Parry, 2023). Testa et al. (2022) discovered that resistance training decreases STAT3 phosphorylation and plasma IL‐6 concentrations in tumour‐bearing mice (Figure 2). Therefore, long‐term aerobic exercise could have myoprotective anti‐inflammatory *and* anti‐catabolic clinical utility for the hearts and skeletal muscles of cachectic patients.

### 5.4 Exercise for the cachectic patient

Standard treatments for CC are lacking due to the complex nature of this syndrome. While the exact mechanisms of muscle wasting are still being elucidated, ongoing research reveals the benefit of pharmacological (e.g., appetite‐stimulants, weight stabilizers) and non‐pharmacological treatments for cancer patients experiencing cachexia, and this area of research is growing rapidly. Recently, exercise has been considered an emerging therapeutic strategy to preserve muscle mass and function, and attenuate inflammation and metabolic dysfunction. Physical activity can easily be adapted to a person's abilities, is cost‐effective, and can be done in a variety of settings. Exercise improves a person's ability to complete their activities of daily living and in the case of CC, this can increase a person's functional capacity and thus their independence. Aerobic and resistance training increase muscle mass, muscle strength and cardiorespiratory fitness – all of which promote an optimal quality of life.

However, despite our growing knowledge, many gaps still exist. The ideal exercise prescription for any cancer patient must be individualized, and this same standard holds for those with CC. The intensity, frequency, duration and type of exercise can have a substantial impact on how the body responds and adapts to exercise, and ongoing research attempts to understand how these components impact skeletal muscle and cardiac muscle mass maintenance. As such, more translational and clinical research must be conducted to fully understand the feasibility and logistical applications of exercise.

## 6 FUTURE DIRECTIONS

A multitude of questions remain regarding the ideal frequency, intensity, duration, volume and type of exercise for the treatment of cardiac and muscle wasting during CC. While some research has investigated exercise intensity (low vs. moderate vs. high) or exercise type (resistance vs. aerobic) to determine how the cachectic phenotype is influenced by exercise, the underlying mechanisms of CC have yet to be elucidated. Preclinical studies continue to contribute to the characterization of the intersection of inflammatory pathways and their regulation of muscle protein balance (i.e., muscle protein synthesis vs. muscle protein degradation) during CC in different tumour models and exercise interventions. Still, more work is needed to fully elucidate the relationship between tumour type, subsequent severity of CC, and affected pathways. Special focus should be placed on distinguishing intricacies, such as specific forms of autophagy (i.e., selective autophagy – mitophagy) which may vary based on tumour type or exercise intervention. Furthermore, more work is needed to translate such findings to the human clinical setting. Such data will be critical in creating tailored prescriptions for exercise as adjuvant therapy in the treatment of CC muscle wasting.

## 7 CONCLUSION

Occurring in up to 50% of cancer survivors, poorly diagnosed, and currently untreatable, CC poses a serious threat to cancer survivors. Despite its complexity, recent research has determined that inflammation plays a central role in the development of CC. Inflammatory cytokines such as TNFα, IL‐6 and IFNγ are major mediators of CC and are known to induce the protein degradative systems responsible for muscle wasting. In this way, inflammation appears to promote a maladaptive upregulation of protein degradation systems: the UPS and the autophagy–lysosome system. Therefore, the interplay between inflammation and protein degradation pathways (ubiquitin–proteasome and autophagy–lysosome) appears to be a critical therapeutic target for controlling CC cardiac and skeletal muscle wasting and dysfunction.

In recent years, exercise has been shown to be beneficial for cancer survivors due to its ability to improve (or maintain) skeletal muscle strength and functional capacity before, during, and/or after anti‐cancer treatment. Therefore, exercise may also be beneficial for cachectic cancer patients. Preclinical studies have shown that exercise attenuates inflammation during CC, primarily by reducing pro‐inflammatory IL‐6 levels and increasing anti‐inflammatory IL‐10. Furthermore, aerobic and resistance exercise mitigates cancer‐induced muscle wasting by reducing protein degradation markers MuRF1, Atrogin‐1, Beclin‐1, p62, FOXO1. As such, exercise could be a promising therapeutic strategy for CC. If implemented early, it could be a powerful preventative strategy to help preserve muscle and function. Unfortunately, as refractory cachexia is approached, exercise therapy becomes progressively less feasible, therefore early diagnosis and early intervention are of utmost importance. Additionally, there is a growing body of preclinical research that indicates that low‐intensity exercise could be sufficient for preserving muscle function during CC. Despite the evidence of preclinical studies, human clinical trials are lacking to assess the feasibility of exercise during CC (Grande et al., 2021). Therefore, more research is needed to determine the influence of exercise frequency, intensity, duration, and volume on the human cachexia phenotype. Additionally, more research is needed to determine how exercise may synergize with other anti‐cancer and anti‐muscle wasting therapies. In summary, leveraging the anti‐inflammatory and anti‐muscle wasting characteristics of exercise may be a valuable tool for the prevention and management of CC.

## AUTHOR CONTRIBUTIONS

All authors have read and approved the final version of this manuscript and agree to be accountable for all aspects of the work in ensuring that questions related to the accuracy or integrity of any part of the work are appropriately investigated and resolved. All persons designated as authors qualify for authorship, and all those who qualify for authorship are listed.

## CONFLICT OF INTEREST

None declared.

## FUNDING INFORMATION

None.
